# The Impact of Resection Margins in Primary Resection of High-Grade Soft Tissue Sarcomas: How Far Is Far Enough?

**DOI:** 10.3390/biomedicines13051011

**Published:** 2025-04-22

**Authors:** Julian Miles Steffens, Tymoteusz Budny, Georg Gosheger, Marieke De Vaal, Anna Maria Rachbauer, Andrea Laufer, Nina Myline Engel, Niklas Deventer

**Affiliations:** 1 Department of Orthopedics and Tumororthopedics, University Hospital Münster, Albert-Schweitzer-Campus 1, 48149 Muenster, Germany; j_stef22@uni-munester.de (J.M.S.); tymoteusz.budny@ukmuenster.de (T.B.); georg.gosheger@ukmuenster.de (G.G.); mariekemathilda.devaal@ukmuenster.de (M.D.V.); anna.rachbauer@ukmuenster.de (A.M.R.); andrea.laufer@ukmuenster.de (A.L.); 2 Department of Orthopedics and Trauma, University Hospital Essen, Hufelandstr. 55, 45147 Essen, Germany

**Keywords:** soft tissue sarcoma, resection margin, local recurrence, distant metastasis, overall survival

## Abstract

**Background/Objectives**: The World Health Organization’s (WHO) classification of tumors contains around 80 entities of soft tissue sarcomas (STSs). Currently, surgery is the standard treatment for patients with localized STS, but the adequacy of resection margins in soft tissue sarcomas (STSs) remains a topic of intense discussion. **Methods**: This single-center study retrospectively reviewed 203 patients with primary high-grade soft tissue sarcoma, including a follow-up period of at least 24 months. Patients with prior resection, secondary STS, metastasis at presentation, or those who required amputational surgery were excluded from the study. Patients were categorized based on their margin thickness: positive (n = 13, 6.4%), 0–1 mm (n = 67, 33.0%), 1–5 mm (n = 70, 34.5%), and >5 mm (n = 27, 13.3%). **Results**: A total of 64 out of 203 (31.5%) patients developed a local recurrence. The estimated 5-year local-recurrence-free survival (LRFS) was 11.5% (CI 4–25%) for positive margins, 58% (CI 51–64%) for margins 0–1 mm, 76% (CI 70–81%) for margins > 1–5 mm, and 93% (CI 88–98%) for margins > 5 mm. No local recurrences occurred in patients with margins > 5 mm and adjuvant radiotherapy. Margin status significantly influenced the development of distant metastasis and overall survival. Adjuvant radiotherapy improved both local control and overall survival. **Conclusions**: To minimize the risk of local recurrence (LR), a resection margin greater than 5 mm should be attained. When adjuvant radiotherapy is applied, the likelihood of LR decreases even more. In scenarios where preserving critical structures is essential, a resection margin of less than 5 mm can be acceptable for ensuring local control.

## 1. Introduction

The World Health Organization’s (WHO) classification of tumors includes approximately 80 entities of soft tissue sarcomas, accounting for less than 1% of all adult solid tumors [1]. Although treatment regimens have shifted toward a more individualized approach, leveraging advanced molecular profiling and thus including immunotherapy and targeted approaches [2], surgery remains the cornerstone treatment for patients with localized STS [3]. In 1980, Enneking et al. [4] categorized resection margins into four grades: intralesional, marginal, wide, and radical. However, due to the qualitative nature of this system, it has become more common to define surgical margins using metric units. According to the 2021 European Society of Medical Oncology (ESMO) Guidelines [3], surgery should feature an “en bloc wide excision with R0 margins”. While there is a consensus on the importance of wide excision for local control of STS [5,6,7,8,9,10,11], there is a debate regarding the optimal margin distance to minimize the risk of local recurrence [7,8,10,11,12] whilst simultaneously trying to preserve the limb and acceptable function. The studies addressing this issue have shown heterogeneous results, often due to the inclusion of low-grade STS and re-resections. According to the literature, prognostic factors for distant metastases and overall survival besides negative margins are even harder to assess, and a consensus is yet to be agreed upon. Although survival rates have gradually improved over the past two decades, progress has been limited due to a lack of novel therapeutic strategies and the challenges of conducting large, homogeneous studies in this rare and diverse group of cancers [13]. Now, new systemic therapies tailored based on histotype and grading have shown promising data, such as the combination of chemotherapy and immunoinhibitors [2,14,15]. Yet, the question of adequate surgical resection remains unanswered. The present study aims to further explore the optimal resection margin to accomplish local control of STSs, and to gain deeper insight into potential risk factors for distant metastases and overall survival.

## 2. Materials and Methods

A retrospective single-center study was conducted on 619 patients diagnosed with high-grade soft tissue sarcomas (STSs) who underwent primary surgery at a specialized sarcoma center between 2008 and 2018. Ethical approval was obtained from the local ethics committee (2023-070-f-S).

### 2.1. Staging and Grading

Prior to surgery, patients received standardized staging methods. MRI images were used to define the size and location of the tumor, and CT scans of the chest served to detect distant metastasis. Open biopsies were performed to determine the tumor’s entity according to the latest WHO classification of Tumours of Soft Tissue and Bone [16]. Histopathological analysis was conducted at the same center. Margin thickness was pathologically assessed using the R-classification system [17], categorized as R0 if the tumor did not reach the intact barrier or resection margins, and R1 if there was microscopic tumor contamination or resection alongside the pseudo-capsule. Tumor grading was conducted using the FNCLCC (Fédération Nationale des Centres de Lutte Contre le Cancer) system.

### 2.2. Follow-Up and Exclusion Criteria

A follow-up of at least 24 months including MRI and CT scans was required (n = 359) if the study’s main endpoint (LR) was not reached earlier. The imaging followed a strict scheme of MRIs of the tumor site as well as a low-dose lung-CT every 3 months for the first 2 years. The intervals were then extended to 6 months and from the 6th year on to 12 months. If patients remained tumor-free for more than 10 years, follow-up was completed. If contaminated margins remained and the patients underwent re-resection (n = 108), they were excluded from this study. Patients presenting with secondary STS, metastasis at presentation (n = 29), or those who required amputational surgery (n = 14) were also excluded. Tumors of the bone such as Ewing-sarcoma or osteosarcoma, tumors which derived from soft tissue but originated from within the bone (n = 5), and gastrointestinal stromal tumors (GISTs) or retroperitoneal STS were not included in this study. In total, the number of eligible patients was n = 203.

### 2.3. Variables

The following tumor characteristics were obtained: entity, resection margin, grading, staging, tumor size, and the depth, meaning subfascial or superficial. Margin width categories were based on postoperative assessment. Further, treatment-associated data were collected from the patient’s file such as neoadjuvant or adjuvant therapy. Comorbidities and risk factors including hypertension, obesity, and smoking were noted. Each patient was discussed separately in a multimodal tumor board consisting of several experts from different subjects. Most commonly, they advised an adjuvant treatment in sarcomas that were great in size, aggressive in nature, or had a close or contaminated resection margin. In a limited number of cases, neoadjuvant therapy was applied, mainly for downsizing tumors close to critical structures (major nerves, vessels, or bone). In tumors that were resected with a margin wider than 10 mm, further therapy was rarely indicated. However, each advice remained an individual decision and was based on the patient’s general health and preferences.

### 2.4. Statistics

The occurrence of local recurrence marked the study’s main endpoint, others being distant-metastasis-free survival (DMFS) and overall survival. For statistical analysis, local-recurrence-free survival, DMFS, and OS were calculated according to the Kaplan–Meier method [18] looking at a time span of 5 years for LRFS and DMFS and 10 years for OS. Significance analysis was performed using the log-rank test for nominal factors or the Cox regression model [19] in a bivariate setting for metric covariates. Predictor variables which obtained significance in univariate analysis were then selected for multivariate analysis (Cox regression). To maximize the patient numbers within subgroups, these factors were further refined through backwards Wald method with an exclusion probability of 0.10. A *p*-value of less than 0.05 was considered statistically significant. The data analysis software used was SPSS Statistics Version: 28.0.1.1.

## 3. Results

Among the 203 eligible patients in our cohort, comprising 107 males and 96 females, the median age at the time of surgery was 60 years (range: 19–91 years). Tumor grades were distributed as FNCLCC grade 2 in 55 cases (27%) and grade 3 in 143 cases (70%), with 5 cases (3%) where eventual tumor grade determination was not possible due to histological subtypes not aligning with the FNCLCC grading scale or grading difficulties. The most prevalent tumor types were liposarcoma, followed by Undifferentiated Pleomorphic Sarcoma and Spindle Cell Sarcoma (Table 1). Local recurrences were observed in 64 patients, and distant metastases were detected in 75 patients. In terms of LR, fibrosarcoma was the most aggressive entity (100%), followed by leiomysarcoma (43%), which also caused the most DM (71%, Table 2). In 13 cases (6%), non-adequate resection with positive margins persisted, while the majority showed negative margins (Table 2).

### 3.1. Prognostic Factors for Local Recurrence (LR)

**Figure 1:**
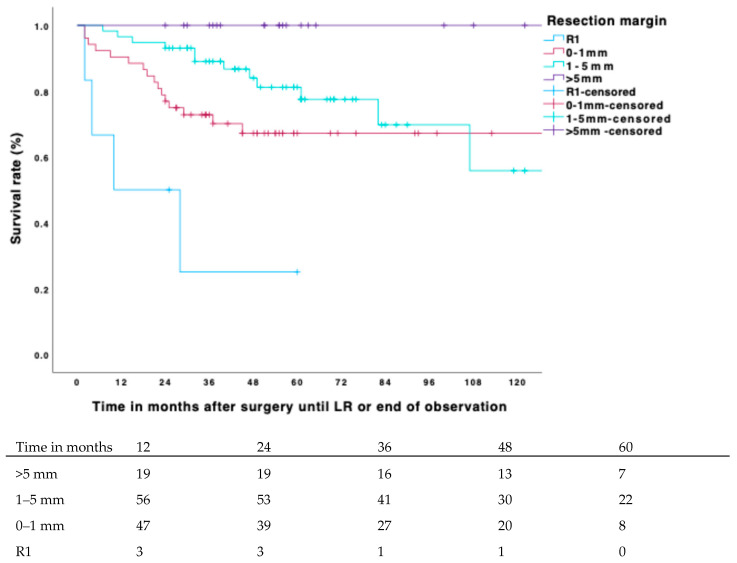
Cumulative incidence of LR for resection margin in patients with adjuvant RTx and number at risk.

**Figure 2:**
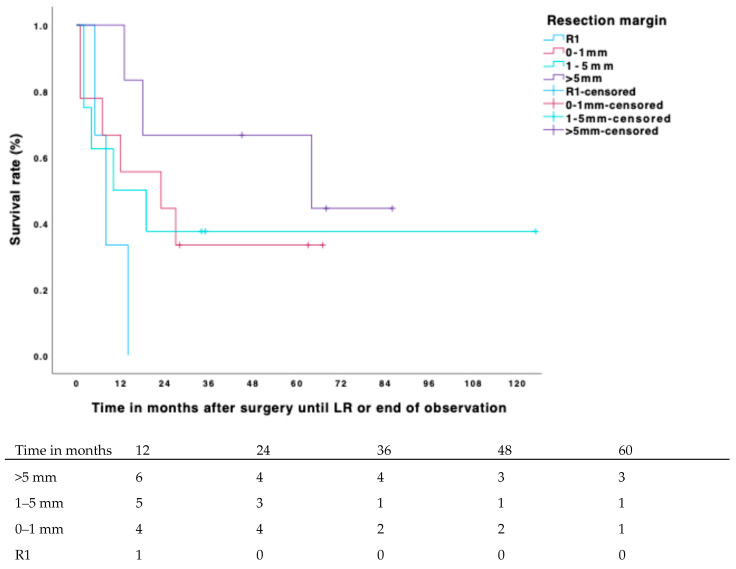
Cumulative incidence of LR for resection margin in patients without adjuvant RTx and number at risk.

The estimated overall 5-year local-recurrence-free survival (LRFS) rate was 70%. Out of 203 patients, 55 (27.1%) developed a local recurrence within this period. The estimated 5-year LRFS for positive margins was 11.5% (CI 4–25%); for margins ≥ 0.1–1 mm, it was 58% (CI 51–64%); for margins > 1–5 mm, it was 76% (CI 70–81%); and for margins > 5 mm, it was 93% (CI 88–98%). Both margin width and adjuvant radiotherapy had a highly significant positive impact on LRFS (Table 3, Figure 1 and Figure 2), with the hazard ratio decreasing as the diameter of the surrounding non-reactive tissue increased. Patients who did not receive radiotherapy had a fourfold higher risk of developing local recurrence compared to those who received adjuvant radiotherapy (*p* < 0.001; HR 0.232; CI 0.126–0.426). Notably, no local recurrences occurred in our study when the margin was >5 mm and adjuvant radiotherapy was applied.

In patients who received adjuvant radiotherapy, the estimated 5-year LRFS was 25% (CI 5–45%) for positive margins, 67% (CI 60–74%) for margins ≥ 0.1–1 mm, 81% (CI 71–87%) for margins > 1–5 mm, and 100% for margins > 5 mm. There was a significant difference in LRFS between patients with resection margins of ≥0–1 mm and >1–5 mm (*p* = 0.07), and this difference showed a trend when adjuvant radiotherapy was applied (*p* = 0.137). Patients with resection margins > 5 mm and adjuvant radiotherapy had a significantly superior outcome compared to those with margins > 1–5 mm and adjuvant radiotherapy (*p* = 0.023). Adjuvant radiotherapy itself had a significant impact throughout the subgroups (R1: *p* = 0.027, >0–1 mm: *p* = <0.001, >1–5 mm: *p* = 0.003, >5 mm: *p* = 0.010). The effect of neoadjuvant therapy was not significant.

No significant differences were found between the R- and UICC-classification systems regarding the cumulative incidence of local recurrence in R0 or R1 resections (*p* = 0.944). Neither tumor grading (*p* = 0.962) nor risk factors such as high blood pressure (*p* = 0.323), smoking (*p* = 0.233), and obesity (*p* = 0.509) influenced LRFS. However, wound-healing complications (*p* = 0.049), chemotherapy (*p* = 0.043), staging (*p* = 0.032), and tumor size (*p* = 0.007) showed a trend toward influencing local control in bivariate analysis but were later excluded through backward selection using the Wald method. In multivariate Cox proportional-hazards regression analysis, age at surgery, adjuvant radiotherapy, and margin width were identified as significant factors (Table 3).

### 3.2. Prognostic Factors for Distant Metastasis (DM)

**Figure 3:**
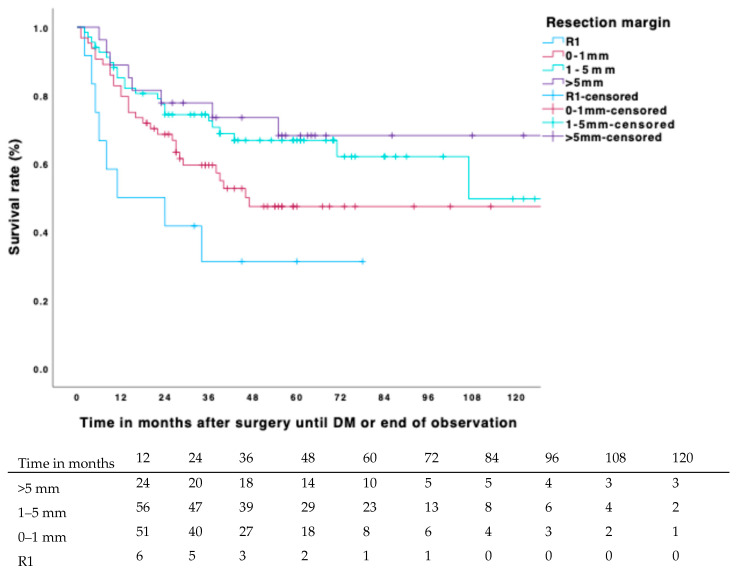
Cumulative incidence of DMFS for margin width and number at risk.

The estimated 5-year DMFS was 60.8% (CI 57–65%); 71 (34.9%) patients developed distant metastases within a five-year span. In bivariate analysis, the covariates’ margin status (*p* = 0.004), margin width (*p* = 0.006), tumor size (*p* = 0.044), local recurrence (*p* <0.001), and chemotherapy (*p* = 0.006) showed significant associations with DMFS, whereas tumor stage and radiotherapy demonstrated trends (*p* = 0.066 and *p* = 0.067, respectively). Notably, when negative resection margins were achieved, the metric resection width no longer significantly influenced metastasis-free survival (*p* = 0.163). Comparing margins greater than 5 mm to margins between 0.1 and 1 mm did not show significant differences in DMFS (*p* = 0.143). The estimated 5-year DMFS rates were as follows: 31.3% (CI: 17–45%) for positive margins, 47.4% (CI: 40–54%) for margins between 0.1 and 1 mm, 66.8% (CI: 61–73%) for margins between >1–5 mm, and 68.2% (CI: 58–78%) for margins greater than 5 mm (Figure 3). In multivariate analysis, both local recurrence (*p* < 0.001; HR 3.288, CI: 1.947–5.551) and adjuvant chemotherapy (*p* = 0.020; HR 0.491, CI: 0.269–0.895) remained significant (Table 4). Adjuvant chemotherapy demonstrated a potential to reduce the risk of developing distant metastasis by more than 2. When LR was removed as an independent covariate in the subsequent Cox regression on the premise that it is closely associated with margin width and adjuvant RTx, margin status became a significant factor (*p* = 0.021, HR 2.475, CI: 1.148–5.336). However, radiotherapy did not exhibit any influence on DMFS in either analysis. Chemotherapy showed similar results when local recurrence was included or excluded as a covariate. Furthermore, no significant changes were observed when analyzing only patients who received adjuvant radiotherapy.

### 3.3. Prognostic Factors for Overall Survival (OS)

Mean OS was 96 months (Range: 2–192 months) with an estimated 10-year overall survival rate of 60% (CI 57–64%). Within this timeframe, 77 (38%) people died, of which 48 (62%) were confirmed tumor-related deaths. The log-rank test and, respectively, the bivariate Cox regression showed a significant correlation for several variables: staging (*p* = 0.002), margin width (*p* = 0.003), margin status, local recurrence, distant metastasis, radiotherapy, and chemotherapy (*p* < 0.001 for each variable), tumor depth (*p* = 0.032), wound-healing complications (*p* = 0.002), duration of surgery (*p* = 0.008), age at surgery (*p* = 0.013) and tumor size (*p* = 0.001).

**Figure 4:**
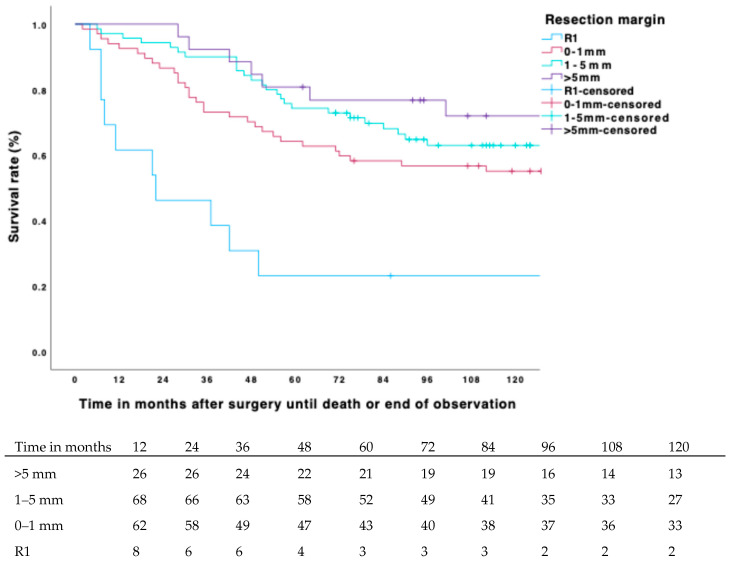
Cumulative incidence of OS for margin width and number at risk.

As with DMFS, if negative margins were achieved, there was no significant correlation between OS and the metrical margin width (*p* = 0.458; Figure 4. Again, the examined risk factors, obesity (*p* 0.889), high blood pressure (0.115), and smoking (0.863), did not appear to contribute to OS.

In multivariate Cox analysis (Table 5), local recurrence (HR 2.765; 1.655–4.620) and distant metastasis (HR 8.705; 4.915–15.418) were identified as the worst prognostic factors decreasing chances of long-time survival by almost 3 and 9 times, respectively. In addition, patients who received either adjuvant radio- (HR 0.482; CI 0.243–0.954) or chemotherapy (HR 0.419, CI 0.213–0.823) showed a superior outcome in terms of OS compared to those who did not receive adjuvant therapy. In contrast, both neoadjuvant radio- and chemotherapy demonstrated a trend but could not significantly improve OS. Wound-healing complication was also identified as an independent prognostic factor for OS (HR 2.247; CI 1.255–4.021). However, when LR was excluded from the Cox analysis, as was done when analyzing DMFS, resection margins and adjuvant radiotherapy gained a stronger influence (*p* = 0.094 and 0.003, respectively) while adjuvant CTx remained almost unchanged.

## 4. Discussion

There is a broad consensus on the fact that positive margins increase the risk of local recurrence [5,6,7,8,9,10,11,12,20,21,22] and that radiotherapy can improve local control [23,24,25,26]. When it comes to an adequate resection margin, however, there is still some dissent. This is mostly due to the rarity of soft tissue sarcomas, which only account for approximately 1% of new malignant neoplasms in adults. Hence, to reach enough cases, studies about STS often encompass heterogenous patient populations with varied tumor entities, grades, and treatments across different institutions and, therefore, different surgeons, procedures, and pathologists. Moreover, they integrate re-resections, whoops procedures, LR, and DM at presentation.

Dickinson et al. categorized [12] 303 patients based on achieved surgical margins: contaminated, <1 mm, but clear, 1–4 mm, 5–9 mm, and 10–19 mm. They concluded that “the margin can safely be as narrow as 1 mm in terms of low local recurrence rates”. However, their study did not consider critical factors such as tumor size and resection difficulty and included previously treated patients. Bilgeri et al. [8] categorized all R0 patients, including those who presented with LR and DM, into four categories, <1 mm, 1–5 mm, 5–10 mm, and >10 mm, and found that wider margins were associated with better outcomes up to 5 mm, beyond which there was no significant improvement in LRFS and overall survival.

In a broader pooled study population including patients with both low- and high-grade STS, McKee et al. [20] demonstrated that those with microscopically positive margins and close negative margins (1–9 mm) were at increased risk for LR and distant metastases compared to patients with clear margins measuring ≥10 mm. Having said this, the study is further limited by its low percentage of adjuvant radiotherapy (38%) and its inclusion of re-resections. In the most selective study, Fujiwara et al. [7] were able to show that in a cohort composed only of patients with infiltrative STS, a surgical margin of greater than 10 mm was associated with an improved LR-free survival. However, these findings are limited because not all negative margins could retrospectively be sorted into the right category [8]. In a prospective study including all types of STS and previously treated patients, Sampo et al. [10] reported that the LRFS correlated with increasing surgical margin as far as 4 cm and indicated that a surgical margin of 2–3 cm provided “reasonable local control”.

In the most recent study published by Yurtbay et al. [11], the authors proposed that “a negative surgical margins distance greater than 1 mm is correlated with a reduced incidence of LR in patients compared to a negative margin distance of less than 1 mm”. While their study design was comparable to ours, the exclusion criteria were not as strict, again including low-grade STS and dividing the subgroups into patients with positive margins and margins lower and greater than 1 mm distance.

Consistent with previous research, our findings demonstrated that negative margins are significantly superior to positive margins, with a hazard ratio (HR) of nearly 8 (HR 7.845; CI 3.984–14.450). Adjuvant radiotherapy also played an important role in achieving local control, reducing the HR of local recurrence (LR) by more than threefold (Table 3). Additionally, our study showed a direct correlation between local-recurrence-free survival (LRFS) and margin width (Figure 1 and Figure 2): larger surrounding non-reactive tissue in the tumor specimen was associated with longer LRFS. Radio- and chemotherapy, as well as tumor stage, were relatively evenly distributed among the subgroups. Patients with resection margins > 5 mm had the best 5-year LRFS at 93%, followed by those with margins of >1–5 mm at 76%. Our 5-year LRFS is slightly lower than most reports in the literature but aligns closely with studies focusing exclusively on high-grade soft tissue sarcomas (STSs), particularly those examining wide resection margins [8,27]. The R0-resection rate of 93.5% in our study compares favorably to published reports that range between 80.7% and 89.8%. In contrast, the local recurrence rate of 31% is on the higher end of prior reports that vary from 11 to 40%. This is most likely due to our strict inclusion criteria, excluding low-grade STS. Notably, in our study, no LR occurred when the margin was >5 mm and adjuvant radiotherapy was applied, a result significantly superior to patients with margins < 5 mm and adjuvant radiotherapy (*p* ≤0.023). Nevertheless, adjuvant radiotherapy improved the LRFS decisively throughout the subgroups and should be applied even in cases in which a resection margin > 5 mm is not feasible due to anatomical barriers or other difficulties.

Age at surgery attained statistical significance in both uni- and multivariate analysis concerning LR, although it only slightly increased the HR. Duration of surgery, considered a composite measure of tumor characteristics such as size, depth, and stage, was significant in bivariate analysis, not aligning with expectations since individual tumor characteristics did not independently reach significance.

It becomes apparent that there is more to predict LRFS than only metric treatment options. Proximity to vascular bundles, nerves, or fascia has become a more critical decision factor in recent years. Kawaguchi et al. [21] first suggested that certain barriers such as the above have a stronger resistance against STS infiltration than normal tissue. O’Donnell et al. [28] emphasized the impact of different clinical settings in patients with positive margins on LRFS and supported the practice of close dissection for the preservation of critical structures even when this leads to microscopically positive margins. They hypothesized that the infiltration of critical structures can hint to a more aggressive nature of the tumor in general. However, this must be weighed against the potential risk of proposing an untrue or falsely assessed surgical barrier which led to the worst 5-year LRFS in their study. Our findings support that the question of what constitutes adequacy should be considered in context but that there is a good chance of achieving local control when a resection margin of more than 5 mm can be achieved, particularly when coupled with adjuvant radiotherapy, which is standard practice in most cases.

While study results are relatively consistent on how resection width and margin status relate to LRFS, an association with DMFS remains questionable [22,29].

It must be said that neither the present study nor one of the studies mentioned above (Table 6) demonstrate any statistically significant correlation between the quantitative resection margin and metastasis-free survival, underscoring the necessity for further research into this topic. Relatively, most DMs were found in leiomyosarcoma (71%), followed by synovial sarcoma (60%). However, both subgroups consisted of low numbers (n = 7 and 15, respectively). While this study could show that margin width and, subsequently, margin status were independent risk factors in univariate analysis, their significance waned in a multivariate setting. In contrast, adjuvant chemotherapy and LR had a decisive impact on DMFS: as shown in the LRFS analyses, there is an inverse relationship between LR and resection width. Analyzing the present study, LR itself proves to be a significant predictor for DM but the causal association has been described before as weak by Trovik et al. [22]. The authors argue that since inadequate margins were the highest risk factor for LR, inadequate margins should also be a significant prognostic factor for DM in an analysis not including LR. However, the authors did not show this in their analysis. Upon removing LR from Cox regression analysis in the present study, margin status assumed critical importance (*p* = 0.021), highlighting its role in influencing DMFS. Radiotherapy, while significant for LR, did not retain significance for DMFS, suggesting a nuanced interplay between treatment modalities and metastatic behavior in STS. Thus, the present study underlines that negative margins are vital in the prevention of both LR and DM. In recent years, novel treatment regimens have emerged, including immunotherapy, targeted therapies, and alternative chemotherapeutic agents beyond the first-line anthracycline-based treatment for advanced STS, demonstrating promising outcomes [2,14,15]. Unfortunately, most of these studies were conducted in patients not eligible for complete resection or in patients with advanced STS, both of which were excluding criteria for this study. Furthermore, since this study primarily focuses on surgical treatment strategies, a detailed investigation of adjuvant therapies is beyond its scope. Therefore, larger-scale studies investigating the interaction between surgical resection margins, (neo)adjuvant therapy, and the biological properties of STS are imperative for devising effective treatment strategies aimed at prolonging DMFS.

Similarly, the impact of margin status on OS remains a topic of debate. While a meta-analysis by Jang et al. [9] and a recent study by Bilgeri et al. [8] concluded that margin status does affect OS, Jang et al. acknowledged several studies that showed contradictory results [29,31,32,33]. However, they acknowledged the low number of participants in the mentioned studies. The latest study conducted by Chouliaras et al. [6] could not find any correlation either but proposed that tumor size and gender are predictive of OS. In another approach, Willeumier et al. argued that due to the aggressive nature of high-grade STS and its decrease in the 5-year survival rate, it is difficult to determine the effect of surgical margin on survival [27] and that the effect will manifest over a longer time on patients who escape early DM [9,24], which is why we set the endpoint for OS at 10 years. While margin status could not uphold its significance in a multivariate setting, the major prognostic factors for OS were LR and DM with an HR of 2.8 and 8.7, respectively. Given the pivotal role of both local recurrence (LR) and distant metastases (DMs) as primary prognostic factors for overall survival (OS), it follows that margin status should significantly influence OS as well. Upon excluding LR and DM in a Cox regression analysis with respect to OS, we observed that R1 resection emerged as a negative prognostic factor (*p* = 0.045, HR 2.209). This underscored the importance of achieving negative margins to optimize OS. Additionally, the findings in the present study suggest that adjuvant radiotherapy and chemotherapy can improve OS, highlighting the potential benefits of adjunctive therapies in enhancing survival outcomes. While specific details on adjuvant radiotherapy and chemotherapy were not collected in this study, optimizing the administration of these therapies akin to Schliemann et al. [34] in R0-resected high-grade STS patients is important.

Interestingly, OS was influenced by wound-healing complications. However, these were mostly noted in patients with positive margins (42%) whereas in other subgroups, only around a fifth of the patients developed wound-healing complications (19%, 21%, 26%). Interestingly, we observed that OS was influenced by wound-healing complications, with a higher incidence noted in patients with positive margins (42%) compared to other subgroups (19%, 21%, 26%). This disparity may be attributed to several factors, including the advanced age of patients with positive margins, their tendency to receive less adjuvant therapy, and the larger average tumor size among patients with wound-healing complications as the tumor might have been in proximity of critical structures.

### Limitations of Our Study

We acknowledge the following limitations in our study. There was an uneven distribution of patients receiving neo- or adjuvant chemotherapy within the subgroups, and the overall numbers were small. Additionally, exact histopathological resection margins were untraceable in 26 patients, limiting our analysis to 177 patients when grouped by resection width. However, when we analyzed by resection status, all 203 patients could be included. To minimize distortions, we established strict inclusion criteria. However, our cohort still included patients with various soft tissue sarcoma (STS) entities, and we could not account for anatomical barriers beyond the superficial or subfascial location of tumors. Furthermore, the retrospective design and the single-center nature of this study reduce its significance regarding general validity, and data collection during the time before treatment in STS has shifted toward a more individualized approach.

## 5. Conclusions

In conclusion, the present study underlines the significance of achieving margins greater than 5 mm, preferably coupled with adjuvant radiotherapy. The highest 5-year local-recurrence-free survival (LRFS) rate was observed in patients meeting these criteria. Moreover, the attainment of negative margins remains important for optimizing distant-metastasis-free survival (DMFS) and overall survival (OS).

## Figures and Tables

**Table 1:** Histological subtypes and grading of the STSs.

Histological Subtype	G2	G3	LR	DM	All
All patients *	55	143	64 (32%)	75 (37%)	203 (100%)
Undifferentiated sarcoma (UPS) **	17	79	34 (35%)	35 (36%)	97 (48%)
Liposarcoma *	18	17	6 (17%)	10 (28%)	36 (18%)
Myxofibrosarcoma	8	16	9 (38%)	6 (25%)	24 (12%)
Synovial sarcoma	5	10	5 (33%)	9 (60%)	15 (7%)
Rhabdomyosarcoma	1	8	3 (33%)	4 (44%)	9 (4%)
Leiomyosarcoma	2	5	3 (43%)	5 (71%)	7 (3%)
Malignant peripheral nerve sheath tumor (MPNST)	0	4	0 (0%)	1 (25%)	4 (2%)
Fibrosarcoma	1	1	2 (100%)	1 (50%)	2 (1%)
Clear cell sarcoma	0	1	0 (0%)	0 (0%)	1 (1%)
Others *	3	2	2 (50%)	4 (50%)	8 (4%)

* numbers may not sum to 100% due to rounding. ** the total may vary from the depicted numbers due to untraceability of resection margin in 26 patients.

**Table 2:** Patient characteristics in total and within subgroups.

Margin Width	Positive	0.1–1 mm	>1–5 mm	>5 mm	Total
Gender					
- Male	6 (46%)	38 (57%)	34 (49%)	18 (67%)	107 (53%)
- Female	7 (54%)	29 (43%)	36 (51%)	9 (33%)	96 (47%)
Age at surgery (median and range)	71 (19–81) years	62 (21–91) years	61 (19–83) years	58 (22–87) years	60 (19–91) years
Duration of surgery (median and range)	173 (85–480) min	114 (30–509) min	99 (39–458) min	105 (34–235) min	105 (30–509) min
Depth					
- Superficial	1 (8%)	7 (11%)	7 (10%)	7 (26%)	25 (13%)
- Subfascial	12 (92%)	56 (89%)	62 (90%)	20 (74%)	169 (87%)
Size of tumor (median)	138 (23–288) mm	103 (22–251) mm	88 (27–301) mm	88 (8–175) mm	96(8–301) mm
Exulcerating					
- Yes	0	2 (3%)	1 (1%)	3 (11%)	6 (3%)
- No	13 (100%)	65 (97%)	69 (99%)	24 (89%)	197 (97%)
Tumor site					
- Right arm	3 (23%)	4 (6%)	4 (6%)	5 (19%)	17 (8%)
- Left arm	1 (8%)	11 (16%)	4 (6%)	1 (4%)	20 (10%)
- Right leg	3 (23%)	30 (44%)	30 (43%)	10 (37%)	81 (40%)
- Left leg	4 (31%)	20 (30%)	28 (40%)	9 (33%)	74 (37%)
- Trunk	2 (15%)	2 (3%)	4 (6%)	2 (7%)	11 (5%)
Tumor stage					
- II	1 (8%)	7 (11%)	10 (14%)	5 (19%)	27 (14%)
- IIIA	3 (23%)	27 (44%)	37 (54%)	13 (48%)	88 (46%)
- IIIB	9 (69%)	28 (45%)	21 (31%)	9 (33%)	76(40%)
Radiotherapy (RTx)					
- Adj.	6 (46%)	52 (78%)	58 (83%)	20 (74%)	155 (76%)
- Neoadj.	4 (31%)	6 (9%)	4 (6%)	1 (4%)	17 (8%)
- No	3 (23%)	9 (13%)	8 (11%)	6 (22%)	31 (15%)
Chemotherapy (CTx)					
- Adj.	-	18 (28%)	25 (37%)	14 (52%)	66 (33%)
- Neoadj.	1 (8%)	2 (3%)	4 (6%)	0 (0%)	9 (5%)
- No	12 (92%)	44 (69%)	39 (57%)	13 (48%)	122 (62%)
Wound-healing complication					
- Yes	5 (42%)	14 (21%)	13 (19%)	7 (26%)	45 (23%)
- No	7 (58%	51 (79%)	56 (81%)	20 (74%)	154 (77%)
Obesity/BMI (kg/m^2^)					
- <18.5	-	1 (2%)	2 (3%)	-	3 (2%)
- 18.5–24.9	5 (39%)	22 (33%)	20 (31%)	7 (28%)	61 (33%)
- 25–29.9	5 (39%)	27 (42%)	23 (36%)	13 (52%)	73 (40%)
- 30–34.9	1 (8%)	11 (17%)	14 (22%)	-	31 (17%)
- 35–39.9	1 (8%)	2 (3%)	2 (3%)	4 (16%)	10 (5%)
- >40	1 (8%)	1 (2%)	3 (4%)	1 (4%)	6 (3%)
Smoking					
- Yes	3 (25%)	14 (22%)	12 (19%)	4 (16%)	36 (20%)
- No	9 (75%)	49 (78%)	50 (81%)	21 (84%)	144 (80%)
High blood pressure					
- Yes	7 (54%)	32 (49%)	29 (45%)	15 (58%)	90 (48%)
- No	6 (46%)	33 (51%)	36 (55%)	11 (42%)	98 (52%)
Local recurrence					
- Yes	11 (85%)	27 (40%)	19 (27%)	4 (15%)	64 (31%)
- No	2 (15%)	40 (60%)	51 (73%)	23 (85%)	139 (69%)
Distant metastasis					
- Yes	8 (67%)	30 (47%)	23 (34%)	9 (33%)	75 (38%)
- No	4 (33%)	34 (53%)	45 (66%)	18 (67%)	121 (62%)
Follow-up (median and range)	13 (3–78) months	37 (1–154) months	45 (3–151) months	55 (24–152) months	46 (1–170) months
Overall survival (median and range)	22 (4–153) months	119 (2–191) months	98 (6–192) months	124 (28–189) months	96 (2–192) months

**Table 3:** Multivariate Cox proportional-hazards analysis for LR.

Covariates	Exp(b)	95% CI of Exp(b)	*p*
Age at surgery	1.017	1.000–1.035	**0.048**
Margin width in mm			
0	**1**		**<0.001**
0.1–1	0.304	0.143–0.646	**0.002**
>1–5	0.178	0.079–0.401	**<0.001**
>5	0.054	0.016–0.183	**<0.001**
Radiotherapy			
No. RTx	**1**		**<0.001**
Neoadj. RTx	1.229	0.564–2.678	0.605
Adj. RTx	0.232	0.126–0.426	**<0.001**

Exp(b), hazards ratio; 95% confidence interval for hazards ratio; *p*, probability *p* (bold ≤ 0.05).

**Table 4:** Multivariate Cox proportional-hazards analysis for DM.

Covariates	Exp(b)	95% CI of Exp(b)	*p*
Margin status			
R0	**1**		0.335
R1	1.477	0.669–3.261	
Radiotherapy			
No RTx	**1**		0.373
Neoadj. RTx	1.439	0.547–3.783	0.164
Adj. RTx	1.735	0.798–3.772	0.461
Chemotherapy			
No CTx	**1**		**0.026**
Neoadj. CTx	1.627	0.635–4.167	0.311
Adj. CTx	0.491	0.269–0.895	**0.020**
Local recurrence			
No	**1**		**<0.001**
Yes	3.328	1.947–5.551	

Exp(b), hazards ratio; 95% confidence interval for hazards ratio; *p*, probability *p* (bold ≤ 0.05).

**Table 5:** Multivariate Cox proportional-hazards analysis for OS.

Covariates	Exp(b)	95% CI of Exp(b)	*p*
Age at surgery	1.018	1.000–1.037	0.056
Margin status			
R0	**1**		0.356
R1	1.482	0.643–3.416	
Radiotherapy			
No RTx	**1**		0.108
Neoadj. RTx	0.555	0.232–1.329	0.186
Adj. RTx	0.482	0.243–0.954	**0.036**
Chemotherapy			
No CTx	**1**		**0.032**
Neoadj. CTx	0.637	0.215–1.888	0.416
Adj. CTx	0.419	0.213–0.823	**0.012**
Local recurrence			
No	**1**		**<0.001**
Yes	2.765	1.655–4.620	
Distant metastasis			
No	**1**		**<0.001**
Yes	8.705	4.915–15.418	
Wound-healing complications			
No			**0.006**
Yes	2.247	1.255–4.021	

Exp(b), hazards ratio; 95% confidence interval for hazards ratio; *p*, probability *p* (bold ≤ 0.05).

**Table 6:** Overview over the literature: resection margins and their impact on LR in STS patients.

Reference	Nr. of Patients	Resection Margin Categories	Impact on LR	Limitations
Dickinson I.C. et al., ANZ J. Surg. 2006 [12]	303	Contaminated, <1, 1–4, 5–9 and 10–19 mm	Margin can safely be as narrow as 1 mm	Including re-resections, regardless of tumor size and difficulty of resection
Bilgeri A. et al., Cancers 2020 [8]	305	Contaminated, <1, 1–5, >5 and >10 mm	A margin of >5 mm is sufficient, wider margins do not benefit the patients	Including re-resections; margin, tumor size, and age are linked to RTx and CTx.
McKee MD et al., J. Surg. Oncol. 2004 [20]	111	Contaminated, 1–9 and >10 mm	Margins > 10 mm are optimal for extremity resections	Including low-grade STS and re-resections, low percentage of adj. RTx and CTx
Fujiwara T et al., Eur. J. Surg. Oncol. 2020 [7]	305	Contaminated, 0.1–0.9, 1.0–1.9, 2.0–4.9, 5.0–9.9 and >10 mm	A margin of >10 mm is advocated	Not all negative margins could retrospectively be sorted into the right WHO category
Kainhofer V et al., Eur. J. Surg. Oncol. 2016 [5]	265	UICC- and R-Classification	R0 resections are superior when classified according to the UICC-classification	Including low- and high-grade STS and re-resections; treatment for atypical liposarcomas changed during the second half
Gundle KR et al., J Clin. Oncol. 2018 [30]	2217	R-Classification, R + 1-Classification and TMCC classification	An R + 1 mm classification reduced LR-differences between R1 and R0, but the R-classification best determined the risk of LR	Single-center study, and treatment protocol has changed over the years; tumor sampling errors cannot be ruled out
Sampo M et al., Br. J. Surg. 2008 [10]	270	<0.4, 0.4–2.0, >2.0 mm	A surgical margin of 2–3 cm provided reasonable local control, even without the use of radiotherapy	Including low- and high-grade STS as well as post-radiation STS and patients who received amputation.
Yurtbay A et al., Medicina 2025 [11]	185	Contaminated, ≤1 and >1 mm	a negative surgical margins distance greater than 1 mm improves LRFS	Including low-grade STS, anatomical boundaries could not be evaluated
Our findings	207	Contaminated, 0.1–1, >1–5 and >5 mm	A margin of >5 mm is advised	Anatomical boundaries could not be evaluated, treatment protocol has shifted over the years

## Data Availability

The datasets used and/or analyzed during the current study are available from the corresponding author on reasonable request.

## Abbreviations

The following abbreviations are used in this manuscript:

WHO	World Health Organization
STS	Soft tissue sarcoma
ESMO	European Society of Medical Oncology
LR	Local recurrence
DM	Distant metastasis
OS	Overall survival
GIST	Gastrointestinal stromal tumor
DMFS	Distant-metastasis-free survival
LRFS	Local-recurrence-free survival
FNCLCC	Fédération Nationale des Centres de Lutte Contre le Cancer
Adj.	Adjuvant
Neoadj.	Neoadjuvant
RTx	Radiotherapy
CTx	Chemotherapy
UICC	International Union against Cancer
AJCC	American Joint Committee on Cancer
TMCC	Toronto margin context classification
CI	Confidence interval
